# Tetra­methyl­ammonium hydrogen terephthalate

**DOI:** 10.1107/S1600536812039487

**Published:** 2012-09-26

**Authors:** Leila Dolatyari, Samad Shoghpour Bayraq, Sara Sharifi, Ali Ramazani, Ali Morsali, Hadi Amiri Rudbari

**Affiliations:** a Department of Chemistry, Zanjan Branch, Islamic Azad University, PO Box 49195-467, Zanjan, Iran; b Department of Chemistry, Faculty of Sciences, Azarbaijan University of Shahid Madani, 35 Km Tabriz-Maragheh Road, PO Box 53714-161, Tabriz, Iran; c Department of Chemistry, Ferdowsi University of Mashhad, Mashhad 91779, Iran; d Department of Chemistry, Faculty of Sciences, Tarbiat Modares University, PO Box 14155-4838, Tehran, Iran; e Department of Chemistry, University of Isfahan, Isfahan 81746-73441, Iran; f Dipartimento di Chimica Inorganica, Vill. S. Agata, Salita Sperone 31, Università di Messina, 98166 Messina, Italy

## Abstract

The asymmetric unit of the title salt, C_4_H_12_N^+^·C_8_H_5_O_4_^−^, contains one half of a tetra­methyl­ammonium cation and one half of a hydrogen terephthalate monoanion. The N atom of the ammonium cation lies on a twofold rotation axis and the centre of mass of the terephthalate anion is on a centre of inversion. In the crystal, the centrosymmetric terephthalate ions are linked by a very short symmetric O—H⋯O hydrogen bond [O⋯O = 2.4610 (19) Å] into a one-dimensional polymeric chain along [1-12]. The tetra­methyl­ammonium cations and terephthalate anions are then connected through a pair of bifurcated acceptor C—H⋯O hydrogen bonds, generating a three-dimensional supra­molecular network. The carboxyl­ate groups at both ends of the terephthalate anion are charge-shared with an equal probability of 0.5.

## Related literature

For a review of very short O—H⋯O hydrogen bonds, see: Speakman (1972 ▶). For recent reports of acidic salts of dicarb­oxy­lic acids with short intra- and inter­molecular O—H⋯O hydrogen bonds, see: Starosta & Leciejewicz (2010 ▶); Hemamalini & Fun (2010 ▶); Sun *et al.* (2002 ▶); Sharma *et al.* (2006 ▶); Wang *et al.* (2004 ▶); Taka *et al.* (1998 ▶). For examples of diphospho­nates with strong O—H⋯O hydrogen bonds, see: Tsaryk *et al.* (2011 ▶); Courtney *et al.* (2006 ▶); Cheng & Lin (2006 ▶). For background to symmetric and asymmetric O—H⋯O hydrogen bonds, see: Misaki *et al.* (1986 ▶); Catti & Ferraris (1976 ▶). For graph-set analysis of hydrogen bonds, see: Etter *et al.* (1990 ▶); Bernstein *et al.* (1995 ▶). For the synthesis of the 5,5′-(*o*-phenyl­ene)di-1*H*-tetra­zole ligand, see: Demko & Sharpless (2001 ▶).
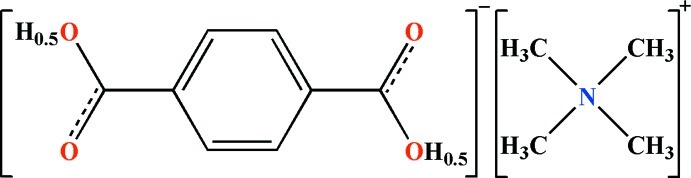


## Experimental

### 

#### Crystal data

C_4_H_12_N^+^·C_8_H_5_O_4_^−^
*M* *_r_* = 239.27
Monoclinic, 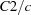

*a* = 16.0585 (4) Å
*b* = 9.1527 (2) Å
*c* = 11.5866 (3) Å
β = 132.915 (2)°
*V* = 1247.21 (7) Å^3^
*Z* = 4
Mo *K*α radiation
μ = 0.10 mm^−1^
*T* = 298 K
0.42 × 0.37 × 0.32 mm

#### Data collection

Bruker APEXII CCD diffractometer
Absorption correction: multi-scan (*SADABS*; Bruker, 2008 ▶) *T*_min_ = 0.708, *T*_max_ = 0.746
20680 measured reflections
1360 independent reflections
1269 reflections with *I* > 2σ(*I*)
*R*_int_ = 0.022

#### Refinement

*R*[*F* ^2^ > 2σ(*F* ^2^)] = 0.054
*wR*(*F* ^2^) = 0.147
*S* = 1.07
1360 reflections
80 parameters
H-atom parameters constrained
Δρ_max_ = 0.57 e Å^−3^
Δρ_min_ = −0.43 e Å^−3^

### 

Data collection: *APEX2* (Bruker, 2007 ▶); cell refinement: *SAINT* (Bruker, 2007 ▶); data reduction: *SAINT*; program(s) used to solve structure: *SHELXS97* (Sheldrick, 2008 ▶); program(s) used to refine structure: *SHELXL97* (Sheldrick, 2008 ▶); molecular graphics: *XPW* (Siemens, 1996 ▶) and *ORTEP-3 for Windows* (Farrugia, 1997 ▶); software used to prepare material for publication: *SHELXTL* (Sheldrick, 2008 ▶) and *enCIFer* (Allen *et al.*, 2004 ▶).

## Supplementary Material

Crystal structure: contains datablock(s) I, global. DOI: 10.1107/S1600536812039487/bg2478sup1.cif

Structure factors: contains datablock(s) I. DOI: 10.1107/S1600536812039487/bg2478Isup2.hkl

Supplementary material file. DOI: 10.1107/S1600536812039487/bg2478Isup3.cml

Additional supplementary materials:  crystallographic information; 3D view; checkCIF report

## Figures and Tables

**Table 1:** Hydrogen-bond geometry (Å, °)

*D*—H⋯*A*	*D*—H	H⋯*A*	*D*⋯*A*	*D*—H⋯*A*
O2—H2⋯O2^i^	1.23	1.23	2.4610 (19)	180 (1)
C8—H8*A*⋯O1^ii^	0.96	2.39	3.267 (3)	152
C9—H9*A*⋯O1^ii^	0.96	2.47	3.321 (3)	148

Symmetry codes: (i) 
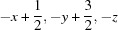
; (ii) 
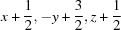
.

**Fig. 1.**
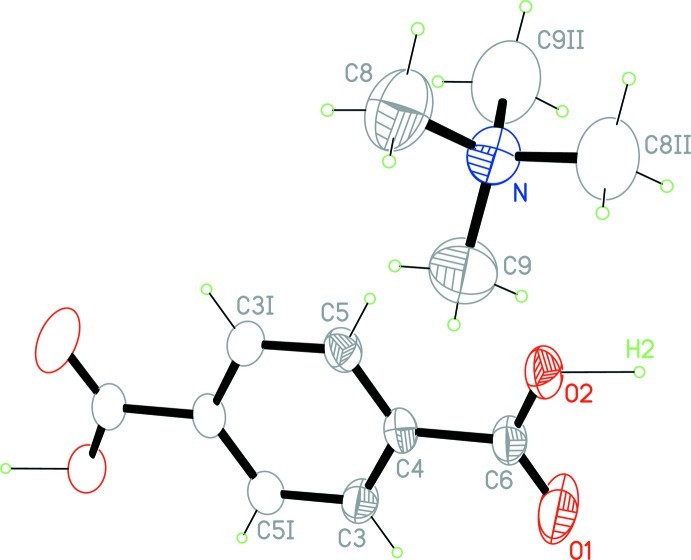
ORTEP drawing of the asymmetric unit of title compound with the atom numbering. Displacement ellipsoids are drawn at the 50% probability level. The occupancy factor for atom H2 is 0.5. Symmetry codes: (i) -x + 1, -y + 2, -z + 1; (ii) -x + 1, y, -z + 1/2.

**Fig. 2.**
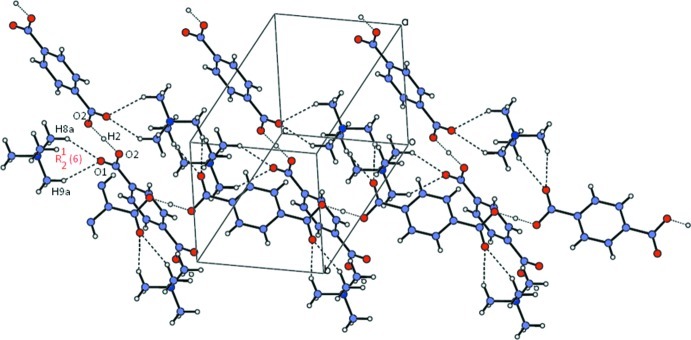
A view of crystal packing of the title compound, showing symmetric O—H···H hydrogen bonds (formed between terephthalate anions, dotted lines) and weak C—H···O hydrogen bonds (formed between cations and anions, dashed lines). The latter contains a pair of bifurcated acceptor hydrogen bonds, leading to an R21(6) ring motif.

## supplementary crystallographic information

### Comment

Acid salts of dicarboxylic acids usually form very short O—H···O hydrogen bonds (with O···O distances between 2.4 and 2.5 Å) in their crystal structures (Speakman, 1972). This type of hydrogen bond is formed between the carboxyl and carboxylate groups by intramolecular (Starosta & Leciejewicz, 2010; Hemamalini & Fun, 2010; Sun *et al.*, 2002) or intermolecular (Sharma *et al.*, 2006; Wang *et al.*, 2004; Taka *et al.*, 1998) interactions. However, diphosphonates can also display such short and strong hydrogen bonds between neighbouring phosphonate groups (Tsaryk *et al.*, 2011; Courtney *et al.*, 2006; Cheng & Lin, 2006). There are two types of short O—H···O hydrogen bonds: symmetric, in which two O atoms are related by crystallographic symmetry and asymmetric, in which crystal symmetry does not impose the O—H···O hydrogen bond to be symmetric. Furthermore, symmetric hydrogen bonds typically display a shorter (2.43–2.51 Å) O···O distance than asymmetric ones (2.44–2.57 Å) (Misaki *et al.*, 1986). In this work, we report the crystal structure of the title compound, whose structure contains a strong symmetric O—H···O hydrogen bond (Catti & Ferraris, 1976): atom H2 lies on a center of symmetry, located between two crystallographic equivalent carboxyl O2 atoms. The H atom, clearly visible on the Fourier map, is involved in a symmetric O2—H2···O2^i^ [symmetry code: (i) -*x* + 1/2, -*y* + 3/2, -*z*] hydrogen bond with an O—H bond distance of 1.23 Å (Table 1).

The title compound (Fig. 1), [N(CH_3_)_4_]^+^[4-COOH-C_6_H_4_COO]^-^, consists of one half tetramethylammonium cation and one half terephthalate anion in the asymmetric unit. The cation lies on a twofold rotation axis and the anion on an inversion center. In the terephthalate anions, the two carboxyl groups are twisted from the mean plane of the benzene ring by a dihedral angle of 6.57 (2)°. Carboxyl atom O2 lies slightly farther [0.083 Å] from this plane than atom O1 [0.065 Å], owing to the strong O—H···O hydrogen bond between the terephthalate anions.

In the crystal, the terephthalate anions are linked end-to-end to form a one-dimensional polymeric chain in which adjacent ions are interconnected by a strong symmetric O—H···O (O···O distance of 2.4610 (19) Å) hydrogen bond (Table 1). Then the weak C—H···O hydrogen bonds link the ammonium cations and terephthalate anions together in a three-dimensional crystal structure. The C8—H8A···O1 and C9—H9A···O1 interactions form a pair of bifurcated acceptor bonds (Fig. 2), involving two C—H donor from an ammonium ion and an acceptor O atom from the terephthalate ion, generating an *R*_2_^1^(6) ring motif (Etter *et al.*, 1990; Bernstein *et al.*, 1995).

### Experimental

In an attempt to synthesize metal–organic framework materials, we obtained the title compound as a side-product.

The ligand H_2_L [5,5'-(*o*-Phenylene)di-1*H*-tetrazole] used in this work was synthesized according to literature procedures (Demko & Sharpless, 2001).

A mixture of Zn(NO_3_)_2_.6H_2_O (0.357 g, 1.2 mmol), H_2_L (0.086 g, 0.4 mmol), terephthalic acid (0.066 g, 0.4 mmol), 1,4-diazabicyclo[2.2.2]octane (0.045 g, 0.4 mmol) and CH_3_OH/DMF (1/2, 15 ml) were sealed in a 25 ml Teflon-lined stainless steel autoclave, heated at 433 K for 40 h, and then cooled to room temperature over a period of 90 h. The resulting solution was filtered and the filtrate was allowed to stand in air at room temperature. After several days, colorless single crystals of the title compound were isolated.

### Refinement

C-H atoms were located on a ΔF map, further idealized and finally refined in the riding model aproximation d(C—H) = 0.93Å; U(H) = 1.2U(C)_eq_; d(C—H_3_) = 0.96Å; U(H) = 1.5U(C)_eq_. Atom H2 is fixed by symmetry, and its isotropic displacement factor was freely refined.

### Crystal data

**Table:**

C_4_H_12_N^+^·C_8_H_5_O_4_^−^	*F*(000) = 512
*M**_r_* = 239.27	*D*_x_ = 1.274 Mg m^−^^3^
Monoclinic, *C*2/*c*	Mo *K*α radiation, λ = 0.71073 Å
Hall symbol: -C 2yc	Cell parameters from 9931 reflections
*a* = 16.0585 (4) Å	θ = 2.8–30.3°
*b* = 9.1527 (2) Å	µ = 0.10 mm^−^^1^
*c* = 11.5866 (3) Å	*T* = 298 K
β = 132.915 (2)°	Irregular, colourless
*V* = 1247.21 (7) Å^3^	0.42 × 0.37 × 0.32 mm
*Z* = 4	

### Data collection

**Table:**

Bruker APEXII CCD diffractometer	1360 independent reflections
Radiation source: fine-focus sealed tube	1269 reflections with *I* > 2σ(*I*)
Graphite monochromator	*R*_int_ = 0.022
φ and ω scans	θ_max_ = 27.0°, θ_min_ = 2.8°
Absorption correction: multi-scan (*SADABS*; Bruker, 2008)	*h* = −20→20
*T*_min_ = 0.708, *T*_max_ = 0.746	*k* = −11→11
20680 measured reflections	*l* = −14→14

### Refinement

**Table:**

Refinement on *F*^2^	Primary atom site location: structure-invariant direct methods
Least-squares matrix: full	Secondary atom site location: difference Fourier map
*R*[*F*^2^ > 2σ(*F*^2^)] = 0.054	Hydrogen site location: inferred from neighbouring sites
*wR*(*F*^2^) = 0.147	H-atom parameters constrained
*S* = 1.07	*w* = 1/[σ^2^(*F*_o_^2^) + (0.0714*P*)^2^ + 1.1167*P*] where *P* = (*F*_o_^2^ + 2*F*_c_^2^)/3
1360 reflections	(Δ/σ)_max_ < 0.001
80 parameters	Δρ_max_ = 0.57 e Å^−^^3^
0 restraints	Δρ_min_ = −0.43 e Å^−^^3^

### Special details

**Table:**

Geometry. All s.u.'s (except the s.u. in the dihedral angle between two l.s. planes) are estimated using the full covariance matrix. The cell s.u.'s are taken into account individually in the estimation of s.u.'s in distances, angles and torsion angles; correlations between s.u.'s in cell parameters are only used when they are defined by crystal symmetry. An approximate (isotropic) treatment of cell s.u.'s is used for estimating s.u.'s involving l.s. planes.
Refinement. Refinement of *F*^2^ against ALL reflections. The weighted *R*-factor *wR* and goodness of fit *S* are based on *F*^2^, conventional *R*-factors *R* are based on *F*, with *F* set to zero for negative *F*^2^. The threshold expression of *F*^2^ > 2σ(*F*^2^) is used only for calculating *R*-factors(gt) *etc*. and is not relevant to the choice of reflections for refinement. *R*-factors based on *F*^2^ are statistically about twice as large as those based on *F*, and *R*-factors based on ALL data will be even larger.

### Fractional atomic coordinates and isotropic or equivalent isotropic displacement parameters (Å^2^)

**Table:**

	*x*	*y*	*z*	*U*_iso_*/*U*_eq_	
O2	0.32784 (9)	0.78319 (13)	0.13940 (12)	0.0451 (4)	
C5	0.51647 (12)	0.89007 (16)	0.43479 (16)	0.0341 (4)	
H5	0.5278	0.8165	0.3914	0.041*	
C4	0.40806 (11)	0.94633 (15)	0.34990 (15)	0.0312 (3)	
C6	0.30731 (12)	0.89045 (17)	0.18691 (16)	0.0363 (4)	
O1	0.21412 (12)	0.94615 (19)	0.11216 (16)	0.0789 (6)	
N	0.5000	0.3608 (2)	0.2500	0.0416 (5)	
C9	0.4511 (2)	0.4550 (3)	0.2947 (3)	0.0794 (8)	
H9A	0.5097	0.5155	0.3823	0.119*	
H9B	0.4188	0.3951	0.3239	0.119*	
H9C	0.3928	0.5157	0.2070	0.119*	
C3	0.39205 (12)	1.05665 (16)	0.41571 (16)	0.0353 (4)	
H3	0.3198	1.0950	0.3594	0.042*	
C8	0.58943 (19)	0.2666 (3)	0.3849 (3)	0.0695 (6)	
H8A	0.6473	0.3266	0.4737	0.104*	
H8B	0.6224	0.2071	0.3565	0.104*	
H8C	0.5565	0.2049	0.4120	0.104*	
H2	0.2500	0.7500	0.0000	0.098 (13)*	

### Atomic displacement parameters (Å^2^)

**Table:**

	*U*^11^	*U*^22^	*U*^33^	*U*^12^	*U*^13^	*U*^23^
O2	0.0364 (6)	0.0526 (7)	0.0300 (6)	−0.0050 (5)	0.0162 (5)	−0.0175 (5)
C5	0.0348 (7)	0.0345 (7)	0.0276 (7)	−0.0003 (5)	0.0191 (6)	−0.0076 (5)
C4	0.0315 (7)	0.0324 (7)	0.0218 (6)	−0.0030 (5)	0.0150 (6)	−0.0043 (5)
C6	0.0319 (7)	0.0405 (8)	0.0235 (6)	−0.0026 (6)	0.0138 (6)	−0.0054 (5)
O1	0.0420 (7)	0.0938 (12)	0.0411 (7)	0.0188 (7)	0.0048 (6)	−0.0263 (7)
N	0.0410 (10)	0.0367 (9)	0.0405 (10)	0.000	0.0252 (9)	0.000
C9	0.0718 (15)	0.0875 (17)	0.0706 (14)	0.0197 (12)	0.0452 (13)	−0.0130 (12)
C3	0.0292 (7)	0.0373 (8)	0.0278 (7)	0.0024 (5)	0.0149 (6)	−0.0043 (5)
C8	0.0627 (13)	0.0587 (12)	0.0605 (13)	0.0133 (10)	0.0315 (11)	0.0174 (10)

### Geometric parameters (Å, º)

**Table:**

O2—C6	1.2739 (19)	N—C8	1.481 (2)
C5—C4	1.390 (2)	C9—H9A	0.9600
C5—C3^i^	1.3896 (19)	C9—H9B	0.9600
C5—H5	0.9300	C9—H9C	0.9600
C4—C3	1.391 (2)	C3—C5^i^	1.3896 (19)
C4—C6	1.5115 (18)	C3—H3	0.9300
C6—O1	1.218 (2)	C8—H8A	0.9600
N—C9^ii^	1.478 (2)	C8—H8B	0.9600
N—C9	1.478 (2)	C8—H8C	0.9600
N—C8^ii^	1.481 (2)		
			
C4—C5—C3^i^	120.25 (13)	N—C9—H9A	109.5
C4—C5—H5	119.9	N—C9—H9B	109.5
C3^i^—C5—H5	119.9	H9A—C9—H9B	109.5
C5—C4—C3	119.41 (12)	N—C9—H9C	109.5
C5—C4—C6	121.22 (12)	H9A—C9—H9C	109.5
C3—C4—C6	119.37 (13)	H9B—C9—H9C	109.5
O1—C6—O2	124.70 (13)	C5^i^—C3—C4	120.33 (13)
O1—C6—C4	119.89 (14)	C5^i^—C3—H3	119.8
O2—C6—C4	115.39 (13)	C4—C3—H3	119.8
C9^ii^—N—C9	108.6 (3)	N—C8—H8A	109.5
C9^ii^—N—C8^ii^	109.57 (15)	N—C8—H8B	109.5
C9—N—C8^ii^	110.17 (15)	H8A—C8—H8B	109.5
C9^ii^—N—C8	110.17 (15)	N—C8—H8C	109.5
C9—N—C8	109.57 (15)	H8A—C8—H8C	109.5
C8^ii^—N—C8	108.7 (2)	H8B—C8—H8C	109.5
			
C3^i^—C5—C4—C3	0.2 (3)	C5—C4—C6—O2	4.7 (2)
C3^i^—C5—C4—C6	179.82 (13)	C3—C4—C6—O2	−175.69 (14)
C5—C4—C6—O1	−176.70 (17)	C5—C4—C3—C5^i^	−0.2 (3)
C3—C4—C6—O1	2.9 (2)	C6—C4—C3—C5^i^	−179.83 (13)

Symmetry codes: (i) −*x*+1, −*y*+2, −*z*+1; (ii) −*x*+1, *y*, −*z*+1/2.

### Hydrogen-bond geometry (Å, º)

**Table:**

*D*—H···*A*	*D*—H	H···*A*	*D*···*A*	*D*—H···*A*
O2—H2···O2^iii^	1.23	1.23	2.4610 (19)	180 (1)
C8—H8*A*···O1^iv^	0.96	2.39	3.267 (3)	152
C9—H9*A*···O1^iv^	0.96	2.47	3.321 (3)	148

Symmetry codes: (iii) −*x*+1/2, −*y*+3/2, −*z*; (iv) *x*+1/2, −*y*+3/2, *z*+1/2.

## References

[bb1] Allen, F. H., Johnson, O., Shields, G. P., Smith, B. R. & Towler, M. (2004). *J. Appl. Cryst.* **37**, 335–338.

[bb2] Bernstein, J., Davis, R. E., Shimoni, L. & Chang, N.-L. (1995). *Angew. Chem. Int. Ed. Engl.* **34**, 1555–1573.

[bb3] Bruker (2007). *APEX2* and *SAINT* Bruker AXS Inc., Madison, Wisconsin, USA.

[bb4] Bruker (2008). *SADABS* Bruker AXS Inc., Madison, Wisconsin, USA.

[bb5] Catti, M. & Ferraris, G. (1976). *Acta Cryst.* B**32**, 2754–2756.

[bb6] Cheng, C.-Y. & Lin, K.-J. (2006). *Acta Cryst.* C**62**, m363–m365. 10.1107/S0108270106024528 16891709

[bb7] Courtney, B. H., Juma, B. W. O., Watkins, S. F., Fronczek, F. R. & Stanley, G. G. (2006). *Acta Cryst.* C**62**, o268–o270. 10.1107/S0108270106009528 16679600

[bb8] Demko, Z. P. & Sharpless, K. B. (2001). *J. Org. Chem.* **66**, 7945–7950. 10.1021/jo010635w 11722189

[bb9] Etter, M. C., MacDonald, J. C. & Bernstein, J. (1990). *Acta Cryst.* B**46**, 256–262. 10.1107/s0108768189012929 2344397

[bb10] Farrugia, L. J. (1997). *J. Appl. Cryst.* **30**, 565.

[bb11] Hemamalini, M. & Fun, H.-K. (2010). *Acta Cryst.* E**66**, o2192–o2193. 10.1107/S160053681003000X PMC3008103 21588569

[bb12] Misaki, S., Kashino, S. & Haisa, M. (1986). *Bull. Chem. Soc. Jpn*, **59**, 1059–1065.

[bb13] Sharma, A., Thamotharan, S., Roy, S. & Vijayan, M. (2006). *Acta Cryst.* C**62**, o148–o152. 10.1107/S0108270106003374 16518053

[bb14] Sheldrick, G. M. (2008). *Acta Cryst.* A**64**, 112–122. 10.1107/S0108767307043930 18156677

[bb15] Siemens (1996). *XPW* Siemens Analytical X-ray Instruments Inc., Madison, Wisconsin, USA.

[bb16] Speakman, J. C. (1972). *Struct. Bond.* **12**, 141–199.

[bb17] Starosta, W. & Leciejewicz, J. (2010). *Acta Cryst.* E**66**, m1561–m1562. 10.1107/S1600536810045903 PMC3011810 21589251

[bb18] Sun, Y.-Q., Zhang, J. & Yang, G.-Y. (2002). *Acta Cryst.* E**58**, o904–o906.

[bb19] Taka, J.-I., Ogino, S. & Kashino, S. (1998). *Acta Cryst.* C**54**, 384–386.

[bb20] Tsaryk, N. V., Dudko, A. V., Kozachkova, A. N. & Pekhnyo, V. I. (2011). *Acta Cryst.* E**67**, o1651–o1652. 10.1107/S1600536811022239 PMC3151912 21837054

[bb21] Wang, Y., Odoko, M. & Okabe, N. (2004). *Acta Cryst.* E**60**, m1178–m1180. 10.1107/S010827010401950X 15467114

